# Focus on nanospace materials

**DOI:** 10.1088/1468-6996/16/5/050301

**Published:** 2015-09-25

**Authors:** Qiang Xu, Kevin C-W Wu, Yusuke Yamauchi

**Affiliations:** 1 National Institute of Advanced Industrial Science and Technology, Japan; 2 Department of Chemical Engineering, National Taiwan University, Taiwan; 3 National Institute for Materials Science, Japan

Nanospace materials, including metal–organic frameworks (MOFs), zeolites, mesoporous materials, and layered compounds, are attracting much attention owing to their high surface area, controllable size and orientation of pores, various framework compositions, and so on. These materials have potential applications in (photo)catalysis, separation, sensing, adsorption and gas storage, and biomedicine (drug delivery systems). Their structure may include inorganic-based materials such as silica, transition-metal oxides and carbons, inorganic–organic hybrid materials, polymers, metals, and so on.

During the past 25 years, we have seen significant advances in the ability to synthesize new nanoporous materials with novel functions, the future for which seems very bright. To realize the full potential of these materials, it is important to control their structure, composition, and morphology. This interdisciplinary research has attracted scientists from various communities, including industry, universities, and smaller research laboratories. This Focus Issue in *Science and Technology of Advanced Materials* aims to highlight recent advances in the design and preparation of different nanospace materials and their applications.

We are grateful to all the involved authors and reviewers, as without their great help this Focus Issue would not have been published on time. While this Focus Issue may not cover all aspects of nanospace materials, it will hopefully ignite new ideas and challenging discussions in the relevant scientific communities.

